# Combined node and link partitions method for finding overlapping communities in complex networks

**DOI:** 10.1038/srep08600

**Published:** 2015-02-26

**Authors:** Di Jin, Bogdan Gabrys, Jianwu Dang

**Affiliations:** 1 School of Computer Science and Technology, Tianjin University, Tianjin 300073, P. R. China; 2 Data Science Institute, Faculty of Science and Technology, Bournemouth University, Poole, Dorset BH12 5BB, UK; 3 School of Information Science, Japan Advanced Institute of Science and Technology, Japan

## Abstract

Community detection in complex networks is a fundamental data analysis task in various domains, and how to effectively find overlapping communities in real applications is still a challenge. In this work, we propose a new unified model and method for finding the best overlapping communities on the basis of the associated node and link partitions derived from the same framework. Specifically, we first describe a unified model that accommodates node and link communities (partitions) together, and then present a nonnegative matrix factorization method to learn the parameters of the model. Thereafter, we infer the overlapping communities based on the derived node and link communities, i.e., determine each overlapped community between the corresponding node and link community with a greedy optimization of a local community function *conductance*. Finally, we introduce a model selection method based on consensus clustering to determine the number of communities. We have evaluated our method on both synthetic and real-world networks with ground-truths, and compared it with seven state-of-the-art methods. The experimental results demonstrate the superior performance of our method over the competing ones in detecting overlapping communities for all analysed data sets. Improved performance is particularly pronounced in cases of more complicated networked community structures.

Many complex systems in the real world exist in the form of networks, such as social networks, biological networks, Web networks, etc., which are collectively referred to as complex networks. One of the main problems in the study of complex networks is the detection of community structure^1^, a subject that keeps attracting a great deal of interest. Although no common definition has been agreed upon, a community within a network is usually defined as a group of nodes that are densely connected with respect to the rest of the network. In the past few years, many different approaches have been proposed to uncover community structure in networks. For good reviews, the interested readers can refer to Ref. 2, 3.

Among the existing community detection methods, the most popular ones belong to the group of methods focusing on the partition of nodes, a.k.a., *node communities*, where communities are disjoint subsets of nodes relatively densely connected within groups but sparsely connected across groups^1^. In this conventional community scheme, a node belongs to only one community. However, it is well known that many real-world networks consist of *overlapping communities* i.e., nodes are members of more than one community^4^. One such example is the numerous communities each of us belongs to, including those related to our scientific activities or personal life (school, hobby, family, and so on). Another example from biology is that a large fraction of proteins simultaneously belong to several protein complexes. Thus, hard clustering is inadequate for the investigation of real-world networks with such overlapping communities. Instead, one requires methods that allow nodes to be members of more than one community in the network.

Various approaches for overlapping community detection have been recently proposed. One of such approaches is based on the idea of clique percolation theory, i.e. that a cluster can be interpreted as the union of small, fully connected subgraphs that share nodes^4^,^5^,^6^. Another type of methods discovers each natural community that overlaps with another by using some local expansion or optimization approaches^7^,^8^,^9^,^10^. The third type of methods, namely the detection of *link communities*, partitions links instead of nodes to discover community structures^11^,^12^,^13^,^14^,^15^,^16^. In link communities a node is considered to overlap with other nodes if the links connected to it belong to more than one cluster. The fourth type of algorithms is based on dynamic label propagation^17^ which has also been extended to overlapping community detection^18^,^19^,^20^. In the label propagation process, each node updates its community belonging coefficients by averaging the coefficients from all its neighbors at each time step; and a parameter *r* is used to control the maximum number of communities with which a node can associate. Besides the above four primary classes of methods, many model-based methods^21^,^22^,^23^,^24^,^25^, which maintain probabilistic community memberships, can also be extended to find overlapping communities. However, this type of methods often requires a threshold for the probabilistic memberships in order to get a community structure, which is difficult to determine for many real applications^3^.

Although there have been several types of algorithms for detecting overlapping communities proposed, finding overlapping community structures more effectively in real and complex networks still poses a formidable challenge. The purpose of this work is to propose a new, more efficient and robust method for finding overlapping communities, the intuitive idea of which is as follows. In node communities, a node belongs to only one community^1^. However, overlapping community structures are ubiquitous in real networks^4^. Forcing a node into one community will fail to accommodate multiple relationships and functions that a node may have, resulting in erroneous representation of the network structure. In link communities, links with a similar relational property form communities so that a node can inherit the community memberships of its adjacent links and, as a result, can naturally belong to multiple communities^11^. However, the link partition typically generates a highly overlapping community structure even though sometimes a network has no overlapping structure at all^26^. This problem stems from the fact that the link partition forces every link into a community while there are real networks that have links that do not fit into any community. To better capture complex organizational structures in real networks, an intuitive idea is that *one should be able to find the best* *overlapping communities between the associated node and link communities*. Here the node and link communities must correspond to each other very well, and hence they should be derived from the same framework.

Based on the above idea, we propose a new method for overlapping community detection. We first describe a stochastic model which accommodates both node and link communities in the same framework; we then present an optimization approach based on nonnegative matrix factorization (NMF) to learn the parameters of the model. Thereafter, we describe a method to infer the overlapping communities from the derived node and link communities of the model, i.e., by determining each overlapped community between the corresponding node and link community with a greedy optimization of a local community function *conductance*^27^. Finally, we introduce a model selection method based on consensus clustering to determine a suitable number of communities.

## Results

In order to assess the performance of our NMF method (described in the Methods section), we have evaluated it on synthetic benchmarks and real-world networks. We also compared it with the following seven state-of-the-art overlapping community detection methods: i) CFinder^4^ which is the most prominent algorithm using clique percolation theory; ii) LFM (Local Fitness Measure)^7^ which is a representative method based on local expansion and optimization; iii) LC (Link Community)^11^ which is the most well-known method for link-community finding; iv) BigClam^25^ which is a recently proposed model-based method which finds overlapping communities using the soft community memberships; v) Oslom^10^ which is a local optimization method with an excellent performance especially on the LFR benchmarks; vi) SVI^16^ which is a very recently proposed model-based method for detecting link communities and, according to the authors, able to handle massive networks; and vii) SLPA^19^ which is a representative algorithm based on a dynamic label propagation process.

These methods have a number of parameters that need to be set. For CFinder, we set the clique size *k* = 4, which returns the best overall results^4^. For LFM, we set *α* = 1, which is a natural choice as it is the ratio of the internal degree to the total degree of the community^7^. For LC and BigClam, we use their default values for the parameters, which are also suggested by the authors^11^,^25^. For Oslom, we use the default of 10 trial optimizations of the lowest hierarchical level, and select the lowest hierarchical level as the resulting partition as suggested by the authors^10^. For SVI, following the guidelines in the paper introducing this method^16^, we assign a link to a community if the approximate posterior probability of a link assignment to a community exceeds a threshold *t*. We take the best NMI values obtained from thresholds *t* = 0.5 and *t* = 0.9. Especially, for experiments on synthetic benchmark networks, we required at least three links of a node to be assigned to a community before assigning the node to that community. For SLPA, as suggested by the authors^19^, we set the maximum number of iterations *T* = 100 and vary parameter *r* from 0.01 to 0.1 for synthetic benchmark networks and from 0.02 to 0.45 for real networks in order to determine its optimal value. The source codes and parameters settings of the methods used here are all obtained from the respective authors.

**Figure 1:**
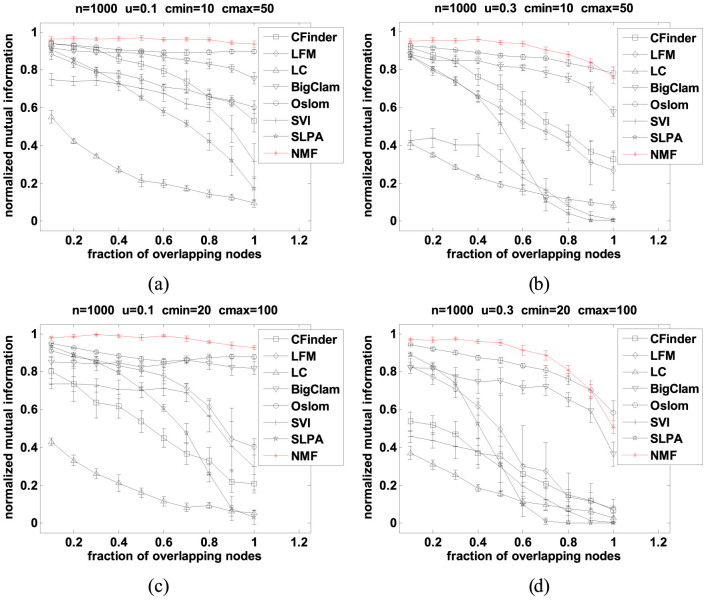
NMI accuracy of each algorithm as a function of the fraction of overlapping nodes. Error bars show the standard deviations estimated from 20 graphs. (a) Comparison on networks with small mixing parameter and small communities (*n* = 1000, *μ* = 0.1, *c_min_* = 10, *c_max_* = 50), (b) Comparison on networks with larger mixing parameter and small communities (*n* = 1000, *μ* = 0.3, *c_min_* = 10, *c_max_* = 50), (c) Comparison on networks with small mixing parameter and larger communities (*n* = 1000, *μ* = 0.1, *c_min_* = 20, *c_max_* = 100), and (d) Comparison on networks with larger mixing parameter and larger communities (*n* = 1000, *μ* = 0.3, *c_min_* = 20, *c_max_* = 100).

**Figure 2:**
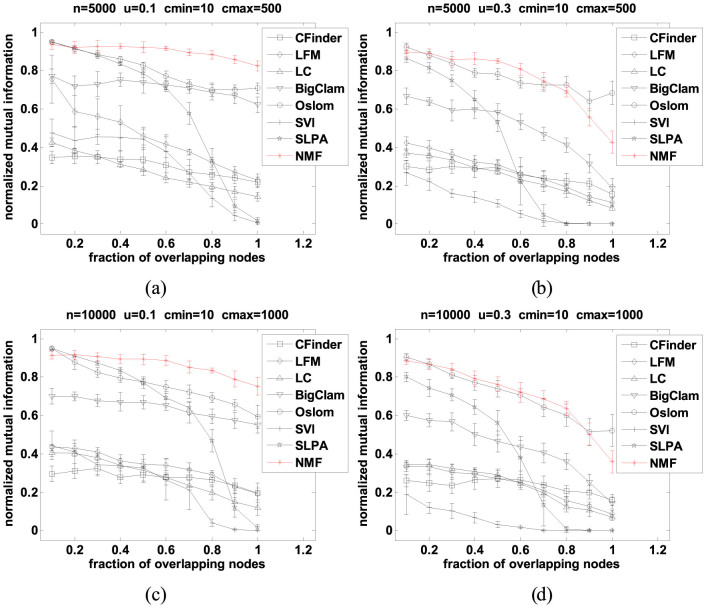
NMI accuracy of each algorithm as a function of the fraction of overlapping nodes on larger networks with more heterogeneous sizes of communities. Error bars show the standard deviations estimated from 20 graphs. (a) Comparison on medium sized networks with small mixing parameter and increased heterogeneity of the sizes of communities (*n* = 5000, *μ* = 0.1, *c_min_* = 10, *c_max_* = 500), (b) Comparison on medium sized networks with larger mixing parameter and increased heterogeneity of the sizes of communities (*n* = 5000, *μ* = 0.3, *c_min_* = 10, *c_max_* = 500), (c) Comparison on the largest networks with small mixing parameter and the largest heterogeneity of the sizes of communities (*n* = 10000, *μ* = 0.1, *c_min_* = 10, *c_max_* = 1000), and (d) Comparison on the largest networks with the largest mixing parameter and the largest heterogeneity of the sizes of communities (*n* = 10000, *μ* = 0.3, *c_min_* = 10, *c_max_* = 1000).

The proposed NMF method requires two hyperparameters, the balance parameter λ and the number of communities *c*, to be provided. In all experiments we use λ = 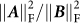 = 1. Two alternative methods to determine λ are described in “Supplementary Information” and experimental results for all three approaches are provided in order to justify our choice. Two different methods, i.e. spectral method^28^ and modularity optimisation method^29^, have been used for finding the initial number of communities required in our model selection procedure for determining the number of communities *c*. Please note that the results for synthetic networks (shown in Figures 1 and 2 and discussed in the section below) are only presented for the spectral method. The modularity optimisation approach was also initially used but as it is know that it tends to generate partitions with communities of very similar sizes it was judged to be not suitable for our experiments with highly heterogeneous sizes of communities which our synthetic networks have (particularly those shown in Figure 2). Both spectral method and modularity optimisation method have been used for the real-world networks experiments.

### Synthetic networks

A type of well-known synthetic benchmarks with overlapping community structure has been proposed by Lancichinetti, Fortunato & Radicchi (LFR)^30^. Here we use it to test the ability of each algorithm to detect known communities under controlled conditions. In the LFR benchmark graphs, both the degree and the community size distributions are power law, which is a statistical property that most real-world networks seem to share.

To quantify the accuracy of community detection methods by evaluating the level of correspondence between detected and ground-truth communities, we employ the widely used normalized mutual information (NMI) index which has been extended to overlapping communities as the accuracy measure^7^. The NMI index, which makes use of information theory, is regarded as a relatively fair metric compared with the other existing metrics^31^ and has therefore been adopted in our study.

Like in the experiment designed by Lancichinetti *et al*^30^, the parameters settings for the first set of LFR benchmarks are as follows. The network size *n* is 1000, the minimum community size *c_min_* is set to either 10 or 20, the mixing parameter *μ* (each vertex shares a fraction *μ* of its edges with vertices in other communities) is set to either 0.1 or 0.3, the fraction of overlapping vertices (*o_n_*/*n*) varies from 0 to 1 with interval 0.1. The remaining parameters which we keep fixed include: the average degree *d* = 20, the maximum degree *d_max_* = 2.5 × *d*, the maximum community size *c_max_* = 5 × *c_min_*, the number of communities each overlapping vertex belongs to *o_m_* = 2, and the exponents of the power-law distribution of vertex degrees *τ*_1_ and community sizes *τ*_2_ are −2 and −1, respectively. This design space leads to four sets of benchmarks.

Figure 1 shows the results that compare our NMF method with CFinder, LFM, LC, BigClam, Oslom, SVI and SLPA in terms of NMI accuracy on the above described LFR benchmark data. As we can see, NMF and Oslom outperform the other 6 methods in all four cases with NMF being even slightly better than Oslom overall. The third most consistently performing method is BigClam. With an increasing fraction of overlapping nodes we can observe a dramatic fall in performance in all the other methods. It is particularly pronounced in case of SLPA which can be quite competitive for small fractions of overlapping nodes but cannot cope with larger fractions of overlapping nodes. Notice that LC and SVI methods do not perform well here. This is because they often find the highly overlapped communities by partitioning links, and fail to detect the communities defined in this benchmark.

To further test the performance of our NMF method, in the second set of experiments, we have increased the size of the networks and increased the ratio of the maximum to minimum sizes of possible communities in the LFR benchmark. To be specific, we first use networks with 5000 nodes and extend the range of community sizes to the interval [10:500] i.e. *c_max_* = 50 × *c_min_*, and then we further increase the size of the networks to 10000 nodes and extend the range of community sizes to the interval [10:1000] i.e. *c_max_* = 100 × *c_min_*. All other graph parameters are the same as in the first set of experiments. This design space has also lead to four sets of benchmarks.

As shown in Figure 2, NMF and Oslom still perform better than the other 6 algorithms in terms of the NMI index on the larger benchmark networks with more heterogeneous sizes of communities. In fact the gap in performance between the proposed NMF method and all the other methods (apart from Oslom) has become wider with a dramatic decrease in performance of CPM, LFM, LC and SVI methods and significant decrease of Bigclam as the size of the network increased (see Figure 2c and 2d in particular). As in the case of the smaller networks (i.e. with 1000 nodes), for the larger networks shown in Figure 2, the SLPA is only competitive for small fractions of overlapping nodes and cannot cope at all with networks with larger fraction of overlapping nodes. Our NMF method has shown particularly good performance and consistently outperformed all the other methods (including Oslam) for smaller value of the mixing parameter *μ* (i.e. *μ* *= * 0.1), larger networks and higher fraction of overlapping nodes. Only for the higher value of the mixing parameter *μ* (i.e. *μ* *= * 0.3) and fraction of overlapping nodes above 0.8 Oslom performed slightly better then NMF (see Figure 2b and 2d).

To sum up, in comparison and contrast to the existing methods on the LFR synthetic benchmarks, the performance of our NMF method is stable and almost not affected by the change of the network size, the heterogeneity in the sizes of communities, the fraction of overlapping vertices, and the ratio of the external degree of each node. The exact reasons for such a good performance are currently under further investigation but we believe that it may be partialy attributed to the proposed approach for overlapping community detection, i.e., finding the best overlapping communities between the associated node and link communities derived from a unified model.

### Real-world networks

As real networks may have some different topological properties that distinguish them from the synthetic ones, we now consider the real-world networks to further compare these methods.

A practical issue in network structure analysis is the lack of the ground-truth of a network. This issue is exacerbated on networks of overlapping structures since overlapping nodes often render ambiguous explanations. Fortunately, there are six real networks with known community structures having been published recently by the Stanford Network Analysis Project^32^. These include four online social networks (LiveJournal, Friendster, Orkut and Youtube), one collaboration network (DBLP) and one information network (Amazon), where the communities, including overlapping ones, in each of these networks are explicitly labeled (see Table 1 for details). Again, we employ the NMI index for overlapping communities as the accuracy measure, so as to consistently evaluate the performance of these algorithms.

The networks used here are very large (see Table 1 for details), which exceeds the capacities of almost all currently available community detection methods. We thus adopted a sampling method to obtain a large set of networks with manageable sizes. Similarly to what was suggested by Yang & Leskovec^25^, we randomly picked a node *u* in the given graph *G* which belongs to at least two communities; we then take the subnetwork to be the induced subgraph of *G* consisting of all the nodes that share at least one known community membership with *u*. Besides, in order to obtain credible subnetworks with well-defined overlapping community structures, for each network we disregard the subnetworks whose values of extended modularity (EQ)^5^ under the ground-truth are less than a threshold of ε = 0.1, which can be considered as having no well-defined community structure^5^. Finally, we generated 500 networks with overlapping communities for each of the 6 datasets in our experiments.

For these real world networks we have also evaluated the impact of two different ways of determining the initial number of communities required by our model selection method (see further details in sections on “Parameter learning” and “Model selection”). In the results below NMF_Spec_ and NMF_Mod_ denote versions of the proposed NMF method for which the initial approximate number of communities in the model selection procedure has been determined by using the spectral method^28^ and modularity optimisation method^29^, respectively.

Quantified by NMI as the performance metric, our NMF method outperformed all the other methods on all six networks (see Table 1). In particular, NMF_Spec_ is 8.99%, 16.03%, 0.60%, 17.16%, 8.27% and 11.51% more accurate and NMF_Mod_ is 10.63%, 15.36%, 2.53%, 14.36%, 10.43% and 15.65% more accurate in terms of NMI values than the second best result from any other non-NMF benchmarked methods on *LiveJournal*, *Friendster*, *Orkut*, *Youtube*, *DBLP* and *Amazon*, respectively. Real-world networks are often known to have more complicated organizational structures than synthetic networks. Given that our method (both NMF_Spec_ and NMF_Mod_) exhibited even better relative performance to all the other methods on the analysed real networks than that reported on synthetic networks, it provides further experimental evidence for the effectiveness of our new idea based on finding the trade-off between and using the complementary information from the node community and link community detection approaches combined in a unified framework. This has resulted in a new approach particularly suitable for complex overlapping structures.

## Discussion

In this work, we propose a novel overlapping community detection method from a new viewpoint that finds the best overlapping communities between the associated node and link communities derived from the same framework. As described in the Methods section, we first describe a unified model that accommodates node and link communities together, and then present a nonnegative matrix factorization method to learn the parameters of the model. Thereafter, in order to infer overlapping communities, we determine each natural community between the corresponding node and link community with a greedy optimization of a local community function conductance^27^. Finally, we use consensus clustering as model selection to determine the number of communities.

We have evaluated our NMF method on both synthetic and real-world networks with ground-truths, and compared it with seven state-of-the-art overlapping community detection methods. The experimental results have demonstrated the superior performance of the NMF over the competing approaches in detecting overlapping communities on the LFR synthetic networks with different network sizes, different heterogeneities of the sizes of communities, different fractions of overlapping vertices, and different ratios of the external degree of each node. Considering real-world networks, a practical issue in the network structure analysis is often the lack of the ground-truth of a network. This issue is exacerbated on networks of overlapping structures since overlapping nodes often render ambiguous explanations. Fortunately, there have been six real networks (including four *online social networks*, one *collaboration network* and one *information network*) with known overlapping community structures published, and thus we have used them to further test our NMF method. Real-world networks are often known to have more complicated organizational structures than synthetic networks, and yet our method exhibited even better relative performance in comparison to all the other evaluated competitive solutions on the examined real networks than that on the synthetic networks. This provides further experimental evidence for the effectiveness of the proposed concept and methods for finding overlapping communities. In the future, we intend to use our NMF method to analyze networks in other fields, but in an attempt to find a balance between the experimental results and not to detract from the main proposed concept, which is the combination of node and link community paradigms within a unified framework, in this paper we have concentrated only on the real networks with available ground truth.

Most community detection methods only make use of information of network topology. Our method as presented in this paper is also an example of such an approach. However, a lot of content on nodes and links is often available in real applications, e.g. Flickr, Facebook and Blog in social media. It is stipulated that the community detection may be significantly improved if one considers this content information, especially when the network has complicated structures or it contains some noise. Several approaches on combining structure and content have already been proposed. Some of them^33^,^34^,^35^,^36^ focused on the incorporation of node content, and some others^37^,^38^ focused on the incorporation of link content. But none of them, to our knowledge, have the ability to make use of all available information. Needless to say, the community structure identification is likely to be greatly benefited by considering both the network topology and node/link content but this seems to be a challenge because if one wants to incorporate the content on both nodes and links one would have to accommodate the community memberships of nodes and links together. Our proposed model is perfectly and, at the moment, uniquely suited for such a task. Thus in the future, we will extend our unified model to incorporate node and link content, so as to even more accurately identify the overlapping communities.

## Methods

In this section, we first describe a stochastic model to accommodate both node and link communities; we then use nonnegative matrix factorization to learn its parameters; thereafter, we describe a method to infer the overlapping communities from the derived node and link communities of the model; and finally, we introduce a model selection method to determine the number of communities.

### Stochastic model

Let *G*(*V*, *E*) be an undirected and unweighted network. The vertex set *V* contains *n* nodes {*v*_1_, *v*_2_, …, *v_n_*}, and the edge set *E* contains *m* edges {*e*_1_, *e*_2_, …, *e_m_*}. Usually, we use the adjacency matrix ***A*** to represent *G*, where *a_ij_* equals to 1 if there is a link between vertices *v_i_* and *v_j_*, and otherwise, it is 0. Besides, we can also use the bipartite graph matrix ***B*** to denote *G*, where *b_ij_* equals to 1 if *v_j_* and *e_i_* are incident, and 0 otherwise. We use *c* probabilistic communities to model the network.

**Figure 3:**
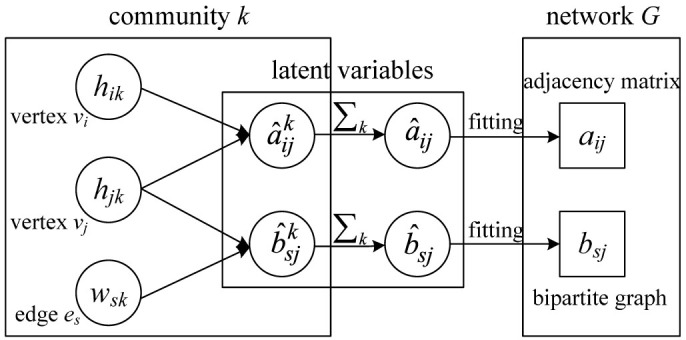
Plate representation of the unified model. We describe node communities ***H*** and link communities ***W*** by fitting the model to network *G* in terms of the adjacency matrix ***A*** and bipartite graph matrix ***B***, respectively.

Our model will have a set of parameters ***H***, where *h_ik_* represents the propensity of vertex *v_i_* belonging to community *k*. We then use ***H*** to generate the expected adjacency matrix 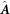 of network *G*. To be specific, *h_ik_h_jk_* is used to denote the expected number of links that lies between vertices *v_i_* and *v_j_* in community *k*. Summing over communities *k*, the expected number of links between *v_i_* and *v_j_* in the network is:
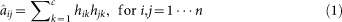
Furthermore, in order to incorporate link communities in the model, we consider another set of parameters ***W***, where *w_ik_* represents the propensity of edge *e_i_* belonging to community *k*. We then use ***W*** and ***H*** to generate the expected bipartite graph matrix 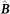. Specifically, *w_ik_h_jk_* is used to denote the expected number of links between *e_i_* and *v_j_* in community *k* in the bipartite graph. Summing over the communities, the expected number of links between *e_i_* and *v_j_* in the bipartite graph is:
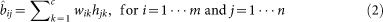
The logistic representation of the entire model is shown in Figure 3, which integrates node and link communities in the same framework. Note that multigraphs and hypergraphs are both allowed here, which is typical for random graph models for simplicity^14^,^38^,^39^.

By using squared loss to measure the relaxation error, our model can be learned by minimizing the following objective function:
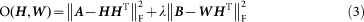
where ||.||_F_ is the Frobenius norm, and ***H*** and ***W*** are nonnegative matrices. The first term denotes the fitting between the expected and actual adjacency matrix of the network; the second term denotes the fitting between the expected and actual bipartite graph matrix of the network; and they are regulated with the use of the balancing parameter λ.

### Parameters learning

According to (3), the learning of the model parameters can be cast as the following optimization problem:
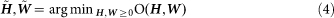
which can be regarded as a problem of nonnegative matrix factorization (NMF). To derive the multiplicative update rule, we adopt a block coordinate descent approach. In particular, the objective function is alternately minimized with respect to ***H*** and ***W***, each time optimizing ***H*** while fixing ***W*** and optimizing ***W*** while fixing ***H***. This way, we decompose the non-convex optimization problem of (4) into two sets of convex subproblems, which are much easier to solve.

Firstly, we derive the update rule of ***H*** while keeping ***W*** fixed. The gradient of (4) with respect to ***H*** can be computed as:
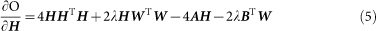
The gradient can be decomposed into a set of positive components and a set of negative components as follows:
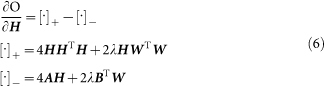
Then, we use the results in Ref. 40 in order to define an iterative learning based update rule with the use of [·]_+_ and [·]_−_ as follows:
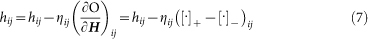
Here *η_ij_* is a positive learning rate. One can choose 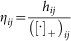, and the update rule becomes a multiplicative update rule:
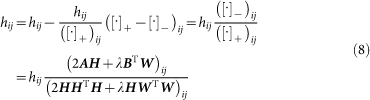
According to the analysis of Ref. 40, ***H*** can be initialized to a nonnegative matrix, and the above multiplicative update rule can be used to maintain nonnegativity. The multiplicative rule converges in the case when ([·]_+_)*_ij_* = ([·]_−_)*_ij_*, which implies that 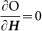 is the stationary point of the objective function.

A similar discussion can be applied to derive the update rule of ***W*** while keeping ***H*** fixed. The gradient of (4) with respect to ***W*** can be calculated as:
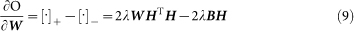
where [·]_+_ and [·]_−_ are respectively the set of positive components and the set of negative components in the gradient. As in the previous case, these can be used in conjunction with the results in Ref. 40 in order to define the following multiplicative update rule:
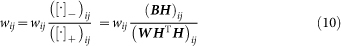
As in the previous case, ***W*** can be initialized to be nonnegative, and the update rule subsequently maintains it. The iterative update of *w_ij_* converges whenever a stationary point 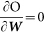 is achieved.

Now, the optimization of (4) is to simultaneously solve (8) and (10), which can be done iteratively by choosing a set of nonnegative initial values and alternating between the two equations. This approach maintains the nonnegativity of the parameters, and monotonically converges to a local minimum of the objective function (corresponding to 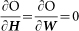).

Besides, our model requires two hyperparameters, the balance parameter λ and the number of communities *c*, to be provided. There are a number of possible ways to determine λ. An intuitive approach is based on the fact that the first and the second terms in the proposed model should have comparable effect on the objective function, if there is no a priori information available to the contrary. Thus in the main part of this paper and comparative analysis with other state-of-the-art methods we set λ = 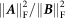 = 1. Two alternative methods to determine λ are described in “Supplementary Information” and experimental results for all three approaches are provided in order to justify our choice. In order to determine the number of communities *c*, we will introduce the model selection method in the following sections. In order to avoid potentially intractable and certainly computationally very expensive exhaustive search for the optimal number of communities as part of our model selection procedure, a heuristic has been proposed and employed which requires an initial approximate number of communities to be given. The spectral method^28^ and the modularity optimisation method^29^ have been used for finding this initial, approximate number of communities and both options have been evaluated and the results presented in the experiments with the real world networks with overlapping communities (see Table 1).

Please take a note that the time to calculate ***AH***, ***B***^T^***W***, ***H***(***H***^T^***H***) and ***H***(***W***^T^***W***) in (8) are 2*mc*, 2*mc*, 2*nc*^2^ and *nc*^2^ +*mc*^2^, respectively, where *n* is the number of nodes, *m* is the number of links and *c* is the number of communities. Thus, the time of evaluating (8) once is *O*(*mc*^2^). The time to calculate ***BH*** and ***W***(***H***^T^***H***) in (10) are 2*mc* and *nc*^2^ +*mc*^2^, respectively, and hence the time of evaluating (10) once is also *O*(*mc*^2^). Therefore, the time complexity of our NMF method is *O*(*Tmc*^2^), where *T* is the iteration number for convergence.

### Inference of overlapping communities

After obtaining the community membership of nodes ***H*** and community membership of links ***W***, the hard partition of nodes (node communities) and hard partition of links (link communities) can be derived as follows. Let *S* = {*S*_1_, *S*_2_,…,*S_c_*} be the hard partition of nodes, in which *S_k_* denotes the *k*-th node community. *S_k_* will be the node set consisting of all nodes *i* satisfying *arg*max*_z_*{*h_iz_* | *z* = 1,2,…,*c*} = *k*. Similarly, Let *R* = {*R*_1_, *R*_2_,…,*R_c_*} be the hard partition of links, in which *R_k_* denotes the *k*-th link community. *R_k_* will be the node set consisting of all links *e_i_* (denoted by its two endpoints <*p*,*q*> = *e_i_*) satisfying *arg*max*_z_*{*w_iz_* | *z* = 1,2,…,*c*} = *k*.

As discussed earlier, node communities force each node into one community, and hence fail to accommodate multiple roles that a node may play. Link communities, on the other hand, force every link into a community while there are background links that should not fit into any community, and thus link based community detection methods typically generate a highly overlapping community structure of nodes. In order to better describe the true community structures, an intuitive idea is that one should be able to find the best overlapping communities between the associated node and link communities such as those derived from our unified model.

In order to infer the overlapping communities *O* = {*O*_1_, *O*_2_,…,*O_c_*} based on the derived node communities *S* = {*S*_1_, *S*_2_,…,*S_c_*} and link communities *R* = {*R*_1_, *R*_2_,…,*R_c_*}, we adopt the following method. We first select a local community function which is suitable for assessing a single community. We then find each *natural community* *O_k_* between *S_k_* and *R_k_* based on the greedy optimization of this objective function. In particular, to detect each community *O_k_*, we make *S_k_* as the seed (*O_k_* = *S_k_*) and take Δ*_k_* = *R_k_* − *S_k_* as the candidate node set. We then iteratively add the node which will bring the highest increase of the community quality of *O_k_* from Δ*_k_* to *O_k_*. This process stops when there is no node in Δ*_k_* that will increase the community quality of *O_k_* when adding this node to it. A summarization of the above method is included in the “Supplementary Information”.

We adopt a well-known local community function, namely *conductance*^27^, as the metric to assess a single community. The conductance of a community *C* can be considered as the ratio between the number of edges within the community and the number of edges between the community nodes and those outside of the community. Formally, the conductance of a community *C* is
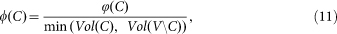
where *φ*(*C*) = |{(*i*, *j*): *i*∈*C*, *j*∉*C*}|, 
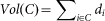
, and *d_i_* is the degree of node *i.* Thus, the lower the conductance of a community, the better it is.

We used *conductance* here due to the fact that it can be efficiently calculated and its good performance in general. However, one may find other community metrics which may be more suitable for specialized applications. Thus in the future, we intend to evaluate and include in our software different community quality metrics.

### Model selection

Recall that our model and NMF method need the number of communities *c* as a hyperparameter to be determined. This is the so-called model selection problem. Similarly to the method proposed by Brunet *et al*^41^, we determine this parameter by exploiting the idea of consensus clustering.

Depending on the random initial conditions our NMF method may or may not converge to the same solution on each run. If a clustering into *k* overlapping communities is strong, we would expect that node assignment to communities would vary only a little from run to run. For each run, the node assignment can be defined by a connectivity matrix ***C_k_*** of size *n* × *n*, with entry ***C_k_***(*i*,*j*) = 1 if nodes *i* and *j* may belong to the same community, and ***C_k_***(*i*,*j*) = 0 if they never belong to the same community, where *k* is the given number of communities. We can then compute the consensus matrix, 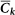, defined as the average connectivity matrix ***C_k_*** over a number of runs (50 runs is generally sufficient to stabilize 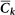). The entries of 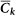 range from 0 to 1 and reflect the probability that nodes *i* and *j* cluster together. If a structure of overlapping communities is stable, we would expect that ***C_k_*** would tend not to vary among runs, and that the entries of 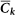 will be close to 0 or 1. Consequently, the general consistency quality of 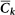 is summarized by the dispersion coefficient defined as
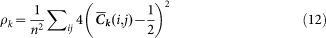
where 0 ≤ *ρ_k_* ≤ 1, and *ρ_k_* = 1 represents a perfectly consistent assignment.

A straightforward way to find the best number of communities is to enumerate all possible *k* to get the one with the maximum *ρ_k_* value^41^. This exhaustive search may become computationally expensive for large networks. Here we offer an alternative to this problem by using an effective heuristic. We first use an assistant community detection method, such as the spectral method^28^ suggested by Darst *et al*^42^, or the widely used though often criticised modularity optimization method^29^ to determine an approximate number of communities *c_s_*. Thereafter, we decrease *k* starting from *c_s_* until *ρ_k_* < *ρ_k_*_ + 1_ and set *c_d_* = *k* + 1, and then increase *k* starting from *c_s_* until *ρ_k_* < *ρ_k_*_ − 1_ and set *c_u_* = *k* − 1. Finally, we determine the best number of communities *c* = *arg*max*_k_* {*ρ_k_*|*k* = *c_d_*,…,*c_u_*}.

Please note that despite wide criticisms in the literature of the modularity optimisation method when used on its own, we have found that it worked well as a method for determining the initial number of communities *c_s_*in the procedure described above and resulted in the best NMI accuracy for four out of six analysed large real world networks.

## Author Contributions

D.J. and B.G. designed the study; D.J., B.G. and J.D. performed the experiments, analyzed the data and prepared the figures; D.J. and B.G. wrote the paper. All authors reviewed the manuscript.

## Supplementary Material

Supplementary Information Supplementary Information: Combined node and link partitions method for finding overlapping communities in complex networks

## Figures and Tables

**Table 1:** Comparison of the NMIs accuracy of different methods on six large Stanford networks with ground-truth of overlapping communities^32^. Here, *n* is the number of nodes, *m* the number of links and *c* the number of communities. M denotes one million and k one thousand. The larger the NMI the better the detected overlapping community structure matches the ground truth available for these networks. The best NMIs for these networks are shown in bold. NMF_Spec_ and NMF_Mod_ represent two versions of NMF method with two different approaches for determining initial approximate number of communities in the NMF model selection procedure

Datasets/NMIs (%)	*n*	*m*	*c*	Methods								
				CFinder	LFM	LC	BigClam	Oslom	SVI	SLPA	NMF_Spec_	NMF_Mod_
LiveJournal	4.0 M	34.9 M	310 k	14.73	14.49	13.84	18.45	22.05	12.22	21.07	31.04	**32.68**
Friendster	120 M	2,600 M	1.5 M	25.26	26.77	18.70	23.30	29.07	17.12	28.96	**45.10**	44.43
Orkut	3.1 M	120 M	8.5 M	14.93	15.60	13.21	18.76	22.92	16.03	25.71	26.31	**28.24**
Youtube	1.1 M	3.0 M	30 k	9.34	13.92	15.81	12.34	13.83	12.98	18.31	**35.47**	32.67
DBLP	0.43 M	1.3 M	2.5 k	13.73	10.84	12.92	14.96	12.16	10.29	12.02	23.23	**25.39**
Amazon	0.34 M	0.93 M	49 k	15.54	14.65	16.28	18.49	17.32	13.65	19.83	31.34	**35.48**
