# Supplementary material for: Combined node and link partitions method for finding overlapping communities in complex networks
Source: Sci Rep. 2015 Feb 26;5:8600. doi: 10.1038/srep08600 (PMC4341207; doi:10.1038/srep08600)
Supplement: Supplementary Information — Combined node and link partitions method for finding overlapping communities in complex networks [file srep08600-s1.pdf]

Supplementary Information:
Combined node and link partitions method for finding overlapping communities in complex networks

Di Jin, Bogdan Gabrys & Jianwu Dang

APPENDIX 1: TWO ALTERNATIVE METHODS TO DETERMINE λ

Here we will discuss how to determine the balance parameter λ and show how it affects the performance of our model. Firstly, we will introduce two alternative methods to determine λ and then compare their results with our original NMF method when setting λ=1.

A1. Learn the Two Terms Separately (LTTS)

The first term of (3) in the main manuscript text is the fitting between the expected adjacency matrix $\hat{\boldsymbol{A}}$ and the actual adjacency matrix $\boldsymbol{A}$ of network G, and hence it can be used alone to describe the node community memberships $\boldsymbol{H}$. The second term of (3) is the fitting between the expected bipartite graph matrix $\hat{\boldsymbol{B}}$ and the actual bipartite graph matrix $\boldsymbol{B}$ of this network. Thus, it can be regarded as a sub-model that describes the relationship between the node community memberships $\boldsymbol{H}$ and link community memberships $\boldsymbol{W}$. Therefore, we divide the model of (3) into two separate parts:

$$\begin{aligned} \mathrm{O}'(\boldsymbol{H}) &= \left\| \boldsymbol{A} - \boldsymbol{H}\boldsymbol{H}^{\mathrm{T}} \right\|_{\mathrm{F}}^{2} \\ \mathrm{O}''(\boldsymbol{W}) &= \left\| \boldsymbol{B} - \boldsymbol{W}\boldsymbol{H}^{\mathrm{T}} \right\|_{\mathrm{F}}^{2} \end{aligned} \tag{A1}$$

which can be regarded as an approximation of the original model, and represents the case when λ=0 in (3). So, we first learn the first sub-model to infer $\boldsymbol{H}$, and then learn the second sub-model based on the derived $\boldsymbol{H}$ to infer $\boldsymbol{W}$. The multiplicative update rules of $\boldsymbol{H}$ and $\boldsymbol{W}$ can be derived separately using the similar methods as discussed in the "Parameters learning" section in the main manuscript text.

We first derive the update rule of $\boldsymbol{H}$. The gradient of $\mathrm{O}'(\boldsymbol{H})$ on $\boldsymbol{H}$ can be calculated as:

$$\frac{\partial \mathrm{O}'}{\partial \boldsymbol{H}} = [\cdot]_{+} - [\cdot]_{-} = 4\boldsymbol{H}\boldsymbol{H}^{\mathrm{T}}\boldsymbol{H} - 4\boldsymbol{A}\boldsymbol{H} \tag{A2}$$

where $[\cdot]_{+}$ and $[\cdot]_{-}$ are respectively the positive and negative terms in the gradient. Then the multiplicative update rule of $\boldsymbol{H}$ can be defined as:

$$h_{ij} = h_{ij}\frac{([\cdot]_{-})_{ij}}{([\cdot]_{+})_{ij}} = h_{ij}\frac{(\boldsymbol{A}\boldsymbol{H})_{ij}}{\left(\boldsymbol{H}\boldsymbol{H}^{\mathrm{T}}\boldsymbol{H}\right)_{ij}} \tag{A3}$$

The optimization of $\mathrm{O}'(\boldsymbol{H})$ is to iteratively solve (A3) by choosing a set of nonnegative initial values. When it converges, we will get the node community memberships $\boldsymbol{H}$.

Similarly, we derive the update rule of $\boldsymbol{W}$ with the given $\boldsymbol{H}$. The gradient of $\mathrm{O}''(\boldsymbol{W})$ on $\boldsymbol{W}$ can be calculated as:

$$\frac{\partial \mathrm{O}''}{\partial \boldsymbol{W}} = [\cdot]_+ - [\cdot]_- = 2\boldsymbol{W}\boldsymbol{H}^{\mathrm{T}}\boldsymbol{H} - 2\boldsymbol{B}\boldsymbol{H} \tag{A4}$$

where $[\cdot]_+$ and $[\cdot]_-$ are the positive and negative terms of the gradient. Then the multiplicative update rule of $\boldsymbol{W}$ can be defined as:

$$w_{ij} = w_{ij}\frac{([\cdot]_-)_{ij}}{([\cdot]_+)_{ij}} = w_{ij}\frac{(\boldsymbol{B}\boldsymbol{H})_{ij}}{\left(\boldsymbol{W}\boldsymbol{H}^{\mathrm{T}}\boldsymbol{H}\right)_{ij}} \tag{A5}$$

Based on the given $\boldsymbol{H}$, the optimization of $\mathrm{O}''(\boldsymbol{W})$ is to iteratively solve (A5) by choosing a set of nonnegative initial values. When it converges, we get the link community memberships $\boldsymbol{W}$.

The time to calculate $\boldsymbol{AH}$ and $\boldsymbol{H}(\boldsymbol{H}^{\mathrm{T}}\boldsymbol{H})$ in (A3) are $2mc$ and $2nc^2$, respectively. Thus, the time of evaluating (A3) once is $O(mc+nc^2)$, and hence the time complexity of getting the optimal $\boldsymbol{H}$ is $O(T(mc+nc^2))$, where T is the iteration number for its convergence. The time to calculate $\boldsymbol{BH}$ and $\boldsymbol{W}(\boldsymbol{H}^{\mathrm{T}}\boldsymbol{H})$ in (A5) are $2mc$ and nc^2+mc^2, respectively. Thus, the time of evaluating (A5) once is $O(mc^2)$, and hence the time complexity of getting the optimal $\boldsymbol{W}$ is $O(Lmc^2)$, where L is the iteration number for its convergence. Therefore, the time complexity of our LTTS method is $O(T(mc+nc^2)+ Lmc^2)$. Please take a note that, although the time complexity of our LTTS method is the same as that of the original method NMF, the LTTS converges much faster because it learns the two terms of (3) separately. Besides, compared with NMF, the LTTS has a much smaller constant coefficient in the time complexity calculation, which makes it even more efficient.

A2. Incorporating the First Term into the Second Term (IFTST)

In order to avoid using λ, we describe another alternative method of formulating our unified model which is based on a linear projection[1]. We first map the adjacency matrix $\boldsymbol{A}$ to the node community membership $\boldsymbol{H}$ with the use of an $n\times c$ transformation matrix $\boldsymbol{X}$, expressed as $\boldsymbol{H}=\boldsymbol{AX}$, which can be taken as an approximation of the first term in (3); we then substitute it to the second term of (3), and thus the variant of our original model can be formulated as:

$$\mathrm{L}(\boldsymbol{X},\boldsymbol{W}) = \left\|\boldsymbol{B} - \boldsymbol{W}(\boldsymbol{A}\boldsymbol{X})^{\mathrm{T}}\right\|_{\mathrm{F}}^2 \tag{A6}$$

In the following, we will use a similar method as discussed in the "Parameters learning" section to learn $\boldsymbol{X}$ and $\boldsymbol{W}$. When the optimal $\boldsymbol{X}$ and $\boldsymbol{W}$ are derived, using $\boldsymbol{H}=\boldsymbol{AX}$ the node and link communities can both be obtained.

We first derive the update rule of $\boldsymbol{X}$ while keeping $\boldsymbol{W}$ fixed. The gradient of (A6) with respect to $\boldsymbol{X}$ can be computed as:

$$\frac{\partial \mathrm{L}}{\partial \boldsymbol{X}} = [\cdot]_+ - [\cdot]_- = 2\boldsymbol{A}^{\mathrm{T}}\boldsymbol{A}\boldsymbol{X}\boldsymbol{W}^{\mathrm{T}}\boldsymbol{W} - 2\boldsymbol{A}^{\mathrm{T}}\boldsymbol{B}^{\mathrm{T}}\boldsymbol{W} \tag{A7}$$

where $[\cdot]_+$ and $[\cdot]_-$ are the positive and negative terms of the gradient. Then the multiplicative update rule of $\boldsymbol{X}$ can be defined as:

$$x_{ij} = x_{ij}\frac{\left([\cdot]_{-}\right)_{ij}}{\left([\cdot]_{+}\right)_{ij}} = x_{ij}\frac{\left(\boldsymbol{A}^{\mathrm{T}}\boldsymbol{B}^{\mathrm{T}}\boldsymbol{W}\right)_{ij}}{\left(\boldsymbol{A}^{T}\boldsymbol{A}\boldsymbol{X}\boldsymbol{W}^{\mathrm{T}}\boldsymbol{W}\right)_{ij}} \tag{A8}$$

Similarly, we derive the update rule of $\boldsymbol{W}$ while keeping $\boldsymbol{X}$ fixed. The gradient of (A6) with respect to $\boldsymbol{W}$ can be calculated as:

$$\frac{\partial \mathrm{L}}{\partial \boldsymbol{W}} = [\cdot]_{+} - [\cdot]_{-} = 2\boldsymbol{W}\boldsymbol{X}^{\mathrm{T}}\boldsymbol{A}^{\mathrm{T}}\boldsymbol{A}\boldsymbol{X} - 2\boldsymbol{B}\boldsymbol{A}\boldsymbol{X} \tag{A9}$$

where $[\cdot]_{+}$ and $[\cdot]_{-}$ are the positive and negative terms of the gradient. Then the multiplicative update rule of $\boldsymbol{W}$ can be defined as:

$$w_{ij} = w_{ij}\frac{\left([\cdot]_{-}\right)_{ij}}{\left([\cdot]_{+}\right)_{ij}} = w_{ij}\frac{\left(\boldsymbol{B}\boldsymbol{A}\boldsymbol{X}\right)_{ij}}{\left(\boldsymbol{W}\boldsymbol{X}^{\mathrm{T}}\boldsymbol{A}^{\mathrm{T}}\boldsymbol{A}\boldsymbol{X}\right)_{ij}} \tag{A10}$$

The optimization of (A6) is to simultaneously solve (A8) and (A10), which can be done iteratively by choosing a set of nonnegative initial values and alternating between the two equations.

The time to calculate $\boldsymbol{A}^{\mathrm{T}}(\boldsymbol{B}^{\mathrm{T}}\boldsymbol{W})$ and $\boldsymbol{A}^{\mathrm{T}}(\boldsymbol{A}\boldsymbol{X})(\boldsymbol{W}^{\mathrm{T}}\boldsymbol{W})$ in (A8) are $4mc$ and mc^2+nc^2+4mc, respectively, and hence the time of evaluating (A8) once is $O(mc^2)$. Besides, the time to calculate $\boldsymbol{B}(\boldsymbol{A}\boldsymbol{X})$ and $\boldsymbol{W}(\boldsymbol{X}^{\mathrm{T}}\boldsymbol{A}^{\mathrm{T}})(\boldsymbol{A}\boldsymbol{X})$ in (A10) are $4mc$ and nc^2+mc^2+4mc, respectively, and thus the time of evaluating (A10) once is also $O(mc^2)$. Therefore, the time complexity of our IFTST method is $O(Tmc^2)$, where T is the iteration number for convergence. Please take a note that, although the time complexity of our IFTST method is the same as that of the original method NMF, the IFTST is more computationally expensive because it has a much larger constant coefficient beyond the time complexity calculation.

A3. Experimental Comparisons

We compare the performance of our original method NMF (λ=1), the fast approximate method LTTS (λ=0) and the linear projection method IFTST ($\boldsymbol{H}=\boldsymbol{A}\boldsymbol{X}$) on the synthetic and real-world networks with ground-truths. The widely-used NMI index for overlapping communities is employed as the accuracy measure[2]. Here we used the spectral method[3] for determining initial approximate number of communities in the NMF model selection procedure.

Similarly to the Experiments section in the main manuscript text, we first test the performance of each algorithm on the LFR benchmarks[4]. Here we only use the artificial networks with 1000 nodes for simplicity. Figure A1 shows the results. As we can see, the performance of LTTS is slightly better than that of NMF, and they both have a higher accuracy than IFTST. Notice that, LTTS has a much smaller solution space than that of the other two methods, and hence it is much easier to converge to an optimal solution. This may be partly the reason why LTTS tends to have a good performance on the LFR benchmarks.

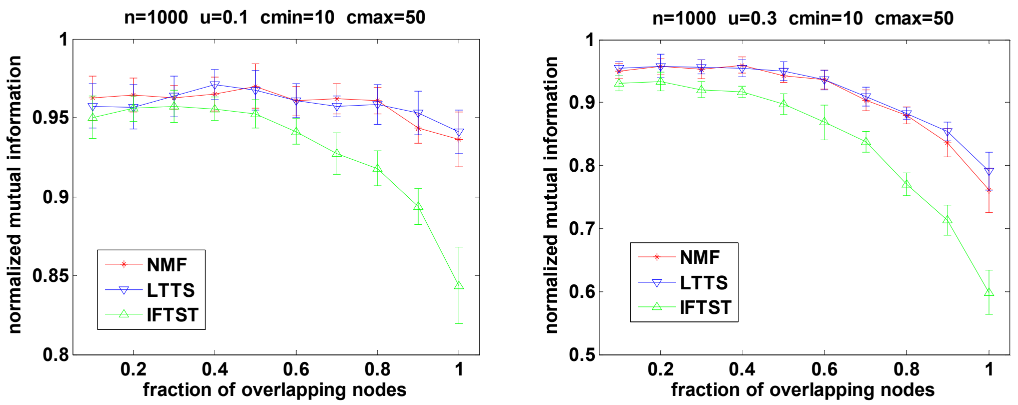

(a) (b)

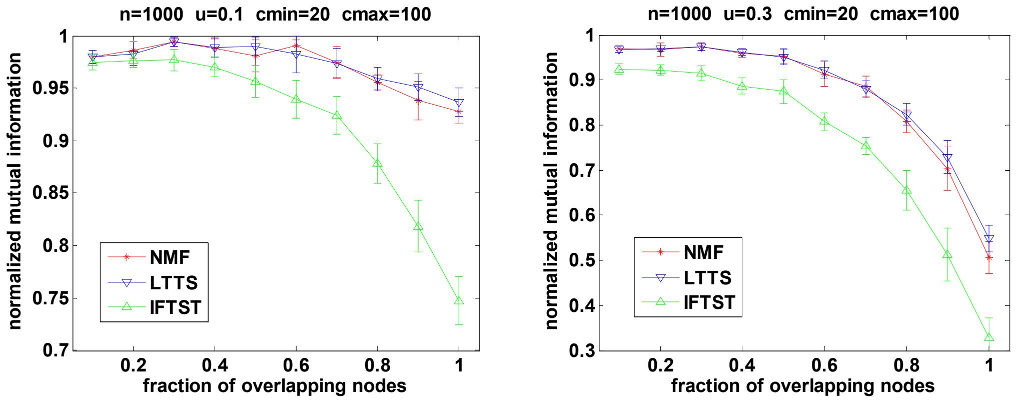

(a) (b)

Figure A1. NMI accuracy of each algorithm as a function of the fraction of overlapping nodes. Error bars show the standard deviations estimated from 20 graphs. **(a)** Comparison on networks with small mixing parameter and small communities (n=1000, μ = 0.1, c_{min} = 10, c_{max} = 50), **(b)** Comparison on networks with larger mixing parameter and small communities (n=1000, μ = 0.3, c_{min} = 10, c_{max} = 50), **(c)** Comparison on networks with small mixing parameter and larger communities (n=1000, μ = 0.1, c_{min} = 20, c_{max} = 100), and **(d)** Comparison on networks with larger mixing parameter and larger communities (n=1000, μ = 0.3, c_{min} = 20, c_{max} = 100).

As in the main manuscript text, we also compare these three methods on six large networks with ground-truth for overlapping communities[5]. With the performance assessed by using NMI as the performance metric, NMF has the best performance on 5 of the 6 networks, and LTTS performs best on the remaining one network. The average accuracy for all six networks of NMF, LTTS and IFTST are 32.08%, 31.82% and 29.98%, respectively. As we can see, on real networks the performance of NMF is slightly better than that of LTTS, and they both perform better than IFTST. This basically matches our previous observation on synthetic benchmarks, i.e., NMF and LTTS are competitive, and they both perform better than IFTST.

Table A1. Comparison of the NMIs accuracy of three proposed methods on six large Stanford benchmark networks. The larger the NMI of an overall community structure, the better the detection of the structure with regard to the available ground truth is. The best NMIs for these networks are shown in bold.

Datasets/ NMIs (%)	Methods		
	NMF	LTTS	IFTST
LiveJournal	**31.04**	31.03	28.13
Friendster	**45.10**	44.78	42.11
Orkut	**26.31**	26.04	25.89
Youtube	**35.47**	35.40	31.33
DBLP	23.23	**23.55**	21.55
Amazon	**31.34**	30.17	30.91

APPENDIX 2: A SUMMARIZATION OF THE METHOD OF INFERING OVERLAPPING COMMUNITIES

The IOC (inference of overlapping communities) method described in the "Inference of overlapping communities" section in the main manuscript text can be summarised as follows:

Algorithm O = IOC (S, R)
For each community k
$O_k = S_k$; $\Delta_k = R_k - S_k$;
While Δ_k is not empty
i= find node $i \in \Delta_k$ that brings the highest increase of O_k's quality when adding i to O_k;
If i is not found
Break;
Endif
$O_k = O_k \cup \{i\}$; $\Delta_k = \Delta_k - \{i\}$;
Endwhile
Endfor

References

1. Qi, G. J., Aggarwal, C. C. & Huang, T. S. Community detection with edge content in social media networks. Paper presented at the 28th IEEE International Conference on Data Engineering, Washington, DC, USA. Piscataway, NJ, USA: IEEE Press. (DOI:10.1109/ICDE.2012.77)(2012, April 1-5).
2. Lancichinetti, A., Fortunato, S. & Kertész, J. Detecting the overlapping and hierarchical community structure in complex networks. *New J. Phys.* **11**, 033015; DOI:10.1088/1367-2630/11/3/033015 (2009).
3. Krzakala, F. et al. Spectral redemption in clustering sparse networks. *Proc. Natl. Acad. Sci. USA* **110**, 20935-20940; DOI:10.1073/pnas.1312486110 (2013).

4. Lancichinetti, A. & Fortunato, S. Benchmarks for testing community detection algorithms on directed and weighted graphs with overlapping communities. *Phys. Rev. E* **80**, 016118; DOI:10.1103/PhysRevE.80.016118 (2009).
5. Leskovec, J. Stanford Network Analysis Project. <http://snap.stanford.edu>, Date of access:11/06/2014.
